# Spermatozoon head size – the main differentiating feature between spermatozoa of blue and white Arctic fox (Vulpes lagopus)

**DOI:** 10.1590/1984-3143-AR2021-0015

**Published:** 2021-10-22

**Authors:** Karolina Stasiak, Dorota Cygan-Szczegielniak, Joanna Bogucka

**Affiliations:** 1 Faculty of Animal Breeding and Biology, Bydgoszcz University of Science and Technology, Mazowiecka, Bydgoszcz, Poland

**Keywords:** arctic fox, ejaculate, sperm dimensions, sperm morphometry

## Abstract

Morphology and sperm morphometry, this is an important determinant of male reproductive capacity. Morphometric data may provide relevant information in studies focused on evolutionary biology, sperm quality assessment, including prediction of the potential fertility, semen cryopreservation, or the effect of reprotoxicants. The paper presents the morphometric analysis of spermatozoa from two colour morphs of Arctic fox (Vulpes lagopus), and attempts to determine the relationship between selected quality indicators and dimensions and shape of spermatozoa. The research material consisted of ejaculates collected once by manual stimulation from 20 one-year-old Arctic foxes (10 individuals of the blue morph and 10 of the white morph). Ejaculates were analysed for standard parameters (volume, sperm concentration, total number of spermatozoa in the ejaculate) and used for the preparation of microscopic specimens. It was found that, the dimensions of spermatozoa from Arctic foxes depend on the male colour morphs. Spermatozoa from white Arctic foxes were significantly longer (by 1.82 µm) and had larger heads (0.32 µm longer and 0.15 µm wider) compared to spermatozoa from blue Arctic foxes (P<0.05). The interactions between particular sperm dimensions indicated the occurrence of gametes differing in shape. The all correlation coefficients between the morphometric traits of spermatozoa were statistically significant. Our research proved that in the blue Arctic foxes, sperm dimensions (tail length and total sperm length) can be related to the percentage of spermatozoa with primary changes (respectively: r = -0.68 and r = -0.75; at P <0.05). However, in the case of white Arctic foxes, these characteristics depend on the ejaculate volume (respectively: r = 0.65 and r = 0.68; at P <0.05).

## Introduction

Reproduction is the most important process of life for all living organisms. In the farms of carnivorous fur animals, it is of particular importance, as they are mostly monestrous species (e.g. foxes, minks). As a result of their monestrous cycle, the offspring from these animals are obtained only once a year. This is why it is so important to choose healthy specimens for reproduction, with semen having the best parameters. This is especially important in the case of young animals (at the beginning of their exploitation) and animals used for insemination, as semen of poor quality may eliminate females from reproduction in a given year. Semen evaluation is essential in predicting male fertility and plays a significant role in maximizing reproductive performance, both in natural conditions and in assisted reproduction (Banaszewska et al., 2015b). Semen quality is most often evaluated by determining the physical properties of ejaculate, namely ejaculate volume, sperm concentration in the ejaculate, and sperm motility. Analysis of sperm morphology is central to the assessment (Oettlé, 1993). In turn, according to Watson (2000), the most important parameter when qualifying sperm for insemination or storage at low temperatures is not only the morphology but also the morphometric characteristics of spermatozoa. Information about spermatozoa size and shape can be treated as a biomarker that identifies a given species or breed. Hence, information about semen from the Arctic fox can be used in the breeding of red foxes (Vulpes vulpes), as well as in the breeding of related species (Soler et al. 2017). However, even spermatozoa characterised by normal morphology may differ in terms of shape. Yániz reported a number of factors that affect the morphometric parameters of spermatozoa. The most important include species, breed, age and sexual maturity of the male, and even handling of the sample and the protocol of morphometric analysis (Yániz et al., 2015). In the case of carnivorous fur animals, sexual maturity coincides with breeding maturity. Arctic foxes reach their full sexual maturity as early as before their first year of life (on average after approximately 9-10 months), and they retain a high reproductive potential for 7 years. As reported by other authors, the sexual development of male mammals does not end with the achievement of sexual maturity or the beginning of reproduction but continues, reaching maximum ejaculation performance at the age immediately preceding somatic maturity (Smital 2009; Banaszewska and Kondracki 2012).

The size and shape of the nucleus and the acrosome surrounding it determine the size and shape of the sperm head, which in turn affects the time necessary for spermatozoa to reach the oocyte and to penetrate the zona pellucida (Buendia et al., 2002 as cited in Banaszewska et al., 2009). According to Malo, spermatozoa with elongated heads are faster than those with rounded heads (Malo et al., 2006). Peña reported that the shape of the sperm head depends on epigenetic factors acting during spermatogenesis (Peña et al., 2005). Research demonstrated that elongated sperm heads may have a deformed nucleus, which in turn can be associated with abnormalities of the connecting piece, presence of the cytoplasmic droplet, and chromatin condensation disorders (Prisant et al., 2007). The precise shape of the sperm head is determined based on strict Tygerberg criteria and four indices: ellipticity, elongation, roughness and regularity (Maree et al., 2010). Ellipticity, expressed as the ratio of sperm head width to length, indicates whether the sperm head is elliptical, thin or conical. The elongation index indicates the degree of rounding of the head, and the roughness index identifies heads with an uneven cell membrane surface, rugose or amorphous. Regularity defines the normality of the sperm head shape and identifies pyriform heads (Czubaszek et al., 2019).

The size of the sperm head, in addition to the head shape, is another important factor in the context of fertilizing capacity. Studies on human sperm have revealed that sperm from fertile men was characterized by smaller heads and lower length/width ratio compared to sperm from infertile individuals or those with reduced fertility (Katz et al., 1986). The results of the experiments on human sperm correspond to the data obtained in veterinary medicine (Casey et al., 1997; Kondracki et al., 2006; Hirai et al., 2001). However, the size of the sperm head is not the only morphological parameter determining successful fertilization. Features of the sperm midpiece and tail are also important. Sperm with longer tails have greater fertilization capacity due to increased motility (their velocity is higher, which means that they can reach the egg cell in a shorter time) (Antończyk, 2012; Fitzpatrick and Lüpold, 2014).

There is a limited number of available studies on the morphometric analysis of sperm from *Canidae*. The significant diversity of canids (according to Nisztuk Pacek, 2016): 16 genera and as many as 41 species) is associated with a high diversity of parameters characterizing ejaculates collected from these animals. Our previous studies indicate that this diversity is not limited to differences between species, but also between colour morphs of the same species (Stasiak et al., 2019). Clear differences in the quality parameters of semen from two colour morphs of the Arctic fox (Vulpes lagopus – formerly known as Alopex lagopus) prompted us to investigate detailed morphometric characteristics of spermatozoa, and to determine the relationship between selected quality indicators and dimensions and shape of spermatozoa.

## Methods

### Animals

Twenty one-year-old male Arctic foxes (Vulpes lagopus) were selected for the study (10 individuals of the blue morph and 10 of the white morph). The experimental foxes were raised in a fur farm where artificial insemination of foxes is practised. All foxes were kept outdoors in roofed cages, one animal per cage. Animals received water at libitum and feed suitable for this species twice daily. The welfare of animals was ensured consistently with the recommendations on the protection of animals kept for farming purposes (European Convention, 1998).

### Semen collection

The experimental material was semen collected at one time (in one day) from selected males in the period of their increased sexual activity, which in the case of the Arctic fox is from mid-February to mid-April. Samples of semen were collected by manual stimulation following the procedure proposed by Kutzler (2005). Ejaculates in which at least 70% of spermatozoa showed progressive movement were included in the analysis. In total 20 ejaculate samples were collected (10 from blue foxes and 10 from white foxes). According to the EU guidelines adopted in Poland (European Union, 2010), this type of research does not require approval from an ethics committee (at the farm semen was regularly collected for the purpose of artificial insemination).

### Semen assessment

**Figure 1:**
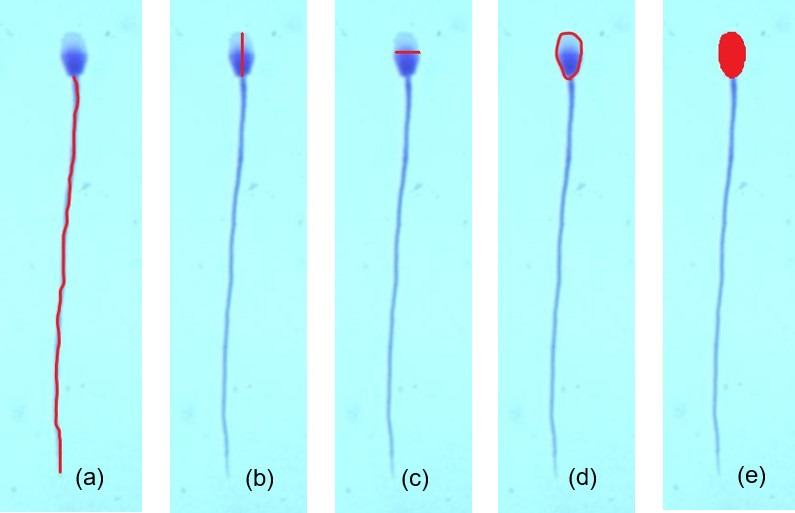
Method of determining sperm dimensions: (a) – tail length, (b) – sperm head length, (c) - sperm head width, (d) – head perimeter, (e) - head area; light microscope, magnification x100.

The collected ejaculates were analysed for volume (measured using a calibrated test tube), sperm concentration (measured by cytometry using the Bürker chamber) and was calculated total number of spermatozoa. Specimens for microscopic analysis were prepared from all ejaculate samples. The method of slide preparation had been described in the previous work by Kondracki et al. (2006). Microscopic examination of the preparations was carried out using immersion objectives at 100x objective magnification and a light microscope Nikon Eclipse 50i (Japan). For each slide we analysed the morphology of 500 spermatozoa, identified sperm with normal and abnormal morphology (spermatozoa with primary and secondary changes). Morphometric tests were carried out employing Screen Measurement v.4.1 (Laboratory Imaging S.r.o. LIM Czech Republic, Praha) for computer analysis of a picture. For each slide, morphometric measurements were taken of 15 randomly selected spermatozoa characterised by normal morphology. Altogether there were taken 300 measurements of spermatozoa. The following morphometric parameters of spermatozoa were analysed: length - L, width – W, perimeter – P, and area – A of the sperm head, tail length, and total sperm cell length (Figure 1). Data from morphometric measurements were used to calculate indices defining the shape of the sperm head: ellipticity (L/W), elongation [(L-W)/(L+W)], roughness [4π(A/P2)], and regularity [π*(L*W/ 4*A)] (Maree et al., 2010).

### Statistical analysis

The results were collated and statistically analyzed using STATISTICA 13.1 software (StatSoft, USA). The data did not comply the assumptions of normality and homogeneity of variance required for parametric statistics. Shapiro-Wilk test and analysis of normal probability graphs showed a non-normal distribution for most variables. The results are presented as median (Q_1_; Q_3_). Mann-Whitney U test was used to determine significant differences between the two groups (blue and white colour morphs males). The interrelationships between the analysed parameters were evaluated based on Spearman's rank correlation coefficients.

## Results

Male blue Arctic foxes produced ejaculates with higher sperm concentration and total number of spermatozoa compared to white Arctic foxes. In blue Arctic foxes sperm concentration was over 192.6x10^6^ spermatozoa per cm^3^ of ejaculate, which was more than 4-fold greater than in ejaculates from white Arctic foxes (47.8x10^6^/cm^3^; P≤0.05). The total number of spermatozoa in samples from blue Arctic foxes was more than 3-fold greater than in ejaculates from white Arctic foxes (respectively: 103.5x10^6^ and 36.1x10^6^; P≤0.05). The analysis did not show a significant difference between ejaculate volume from blue and white colour morphs. The mean volume of ejaculates collected from both morphs of Arctic fox was comparable (blue: 0.71 cm^3^; white: 0.79 cm^3^). The frequency of morphological changes in spermatozoa was quite high. The total percentage of spermatozoa with morphological changes was 22.8% (major abnormalities - 10,7% and minor abnormalities - 12,1%) in sperm from blue Arctic foxes and 22.1% (major abnormalities – 16.0% and minor abnormalities – 6.1%) in white Arctic foxes. Statistical analysis did not reveal significant differences in sperm morphology between blue and white Arctic foxes.

Morphometric parameters of spermatozoa from blue and white Arctic foxes are presented in Table 1. Statistical analysis revealed significant differences in the size of sperm between the two colour morphs. Spermatozoa from white Arctic foxes were significantly longer (by 1.82 µm) and had larger heads (0.32 µm longer and 0.15 µm wider) compared to spermatozoa from blue Arctic foxes (P<0.05). Calculated morphometric indexes allowed for a more precise analysis of sperm head morphology. Sperm collected from both colour morphs of Arctic fox had elliptical (ellipticity index: blue - 1.69, white - 1.70), slightly rounded (elongation: blue, white - 0.26) and symmetrical heads (regularity: blue - 1.02, white - 1.04) with rough of the cell membrane surface (roughness: blue - 0.91, white - 0.89).

**Table 1:** Morphometric characteristics of spermatozoa from blue and white Arctic foxes. The values are presented as median.

**Variable**	**Colour morph**		**P- value**
	**blue n=10**	**white n=10**	
**Tail length (μm)**	65.15a (62.95; 67.94)	66.67b (63.94; 69.55)	0.00396
**Sperm head length (μm)**	6.29^a^ (6.00; 6.61)	6.61^b^ (6.29; 6.92)	0.00000
**Sperm head width (μm)**	3.76^a^ (3.50; 3.96)	3.91^b^ (3.56; 4.17)	0.00205
**Total sperm length (μm)**	71.50^a^ (69.26; 74.43)	73.32^b^ (70.51; 76.21)	0.00044
**Head perimeter (μm)**	15.79^a^ (15.18; 16.41)	16.53^b^ (15.70; 17.34)	0.00000
**Head area (μm^2^)**	18.61^a^ (17.02; 20.04)	19.96^b^ (18.10; 22.41)	0.00000
**Ellipticity**	1.69 (1.58; 1.79)	1.70 (1.61; 1.82)	0.15524
**Elongation**	0.26 (0.22; 0.28)	0.26 (0.23; 0.29)	0.15524
**Regularity**	1.02 (0.95; 1.11)	1.04 (0.97; 1.10)	0.34655
**Roughness**	0.91 (0.85; 0.99)	0.89 (0.85; 0.95)	0.16886

Q_1_-first (lower) quartile - the value of the variable below which 25% of all observations are located; median - second quartile; Q_3_-third (upper) quartile - the value of the variable below which 75% of all observations are located; ^a, b^ P<0.05; n- number of ejaculates; p- probability level.

Correlation coefficients between the physical traits of the ejaculates from Arctic fox and morphometric parameters of spermatozoa are presented in Table 2. The analysis shows that in the blue Arctic foxes, sperm dimensions (tail length and total sperm length) can be related to the percentage of spermatozoa with primary changes (respectively: r = -0.68 and r = -0.75; at P <0.05). However, in the case of white Arctic foxes, these characteristics depend on the ejaculate volume (respectively: r = 0.65 and r = 0.68; at P <0.05).

**Table 2:** Spearman’s coefficients of correlation between the morphometric traits and frequency of development anomalies of spermatozoa and the physical traits of ejaculates.

		**Tail length**	**Sperm head length**	**Sperm head width**	**Total sperm length**	**Head perimeter**	**Head area**
**Blue Arctic fox**	**Sperm concentration**	0.20	0.33	0.13	0.19	0.33	0.36
	**Ejaculate volume**	-0.43	-0.16	0.08	-0.36	-0.20	-0.18
	**Percentage of normal spermatozoa**	0.43	0.32	0.32	0.45	0.30	0.25
	**Percentage of spermatozoa with primary changes**	-0.68*	-0.39	-0.48	-0.75*	-0.58	-0.62
	**Percentage of spermatozoa with secondary changes**	-0.14	-0.23	-0.30	-0.16	-0.16	-0.10
**White Arctic fox**	**Sperm concentration**	-0.14	0.15	-0.12	-0.05	0.16	0.25
	**Ejaculate volume**	0.65^*^	0.46	-0.29	0.68*	0.21	-0.01
	**Percentage of normal spermatozoa**	0.13	0.20	-0.01	0.12	0.33	-0.31
	**Percentage of spermatozoa with primary changes**	-0.21	0.03	-0.07	-0.16	-0.04	0.12
	**Percentage of spermatozoa with secondary changes**	-0.01	0.08	-0.01	0.04	-0.07	0.07

* P<0.05.

The correlation coefficients between the morphometric traits of spermatozoa are presented in Table 3. The most of correlation coefficients between sperm dimensions (in both blue and white Arctic foxes) were statistically significant.

**Table 3:** Coefficients of correlation between the morphometric traits of spermatozoa from blue and white Arctic foxes.

		**Tail length**	**Sperm head length**	**Sperm head width**	**Total sperm length**	**Head perimeter**	**Head area**
**Blue Arctic fox**	**Tail length**	-	0.05	0.17*	0.99**	0.15	0.17*
	**Sperm head length**	0.05	-	0.38**	0.17*	0.87**	0.77**
	**Sperm head width**	0.17*	0.38^**^	-	0.21*	0.76**	0.86**
	**Total sperm length**	0.99**	0,17^*^	0.21*	-	0.25*	0.25*
	**Head perimeter**	0.15	0.87**	0.76**	0.25*	-	0.98**
	**Head area**	0.17*	0.77**	0.86**	0.25*	0.98**	-
**White Arctic fox**	**Tail length**	-	0.17	0.11	0.99**	0.16	0.14
	**Sperm head length**	0.17	-	0.45**	0.26*	0.86**	0.77**
	**Sperm head width**	0.11	0.45**	-	0.16	0.83**	0.91**
	**Total sperm length**	0.99**	0,26*	0.16	-	0.24*	0.22*
	**Head perimeter**	0.16	0.86**	0.83**	0.24*	-	0.98**
	**Head area**	0.14	0.77**	0.91**	0.22*	0.98**	-

* P<0.05; **P<0.001.

## Discussion

Some morphometric data obtained in our study for specific elements of sperm are partly consistent with data reported by Andraszek et al., (2016), who investigated the morphometric features of spermatozoa from silver fox (Vulpes vulpes). Compared to silver fox, sperm from Arctic fox had thinner heads (by about 0.63 μm for the blue colour morph and by 0.48 μm for the white colour morph). In Arctic foxes, sperm heads were more elliptical and less rugose compared to sperm heads from silver fox (Andraszek et al., 2016). Clear differences in the dimensions of sperm cells support the claim that sperm dimensions differ significantly, not only between breeds or species, but also between males representing the same species (Kondracki et al., 2005; Morrow and Gage, 2001). Banaszewska reported, for example, that a single sample of ejaculate may contain sperm of different shapes, sizes and forms (Banaszewska et al., 2015a). This hypothesis was further confirmed by Soler et al. (2017), who analysed kinematic and morphometric parameters of sperm from blue Arctic fox and identified three morphometric subpopulations. According to them, sperm with small and short heads (38.1%) or with large and elliptical heads (35.3%) are identified most frequently in ejaculates, while sperm with thin medium-size heads are found in the lowest number. The results obtained in this study indicate considerable differences in the morphological structure of fox spermatozoa. In the analysis population, spermatozoa with long and wide heads, could be found (the positive correlation between the head length and head width of spermatozoa: blue r=0.38; white r=0.45 at P<0.001). It was also found that the head area of the sperm depends both on the sperm head length (blue r = 0.77; white r = 0.77 with P <0.001) and the sperm head width (blue r = 0.86; white r = 0.91 at P <0.001). According to Hingst, the differences in the dimensions and shape of the head were caused by the variability of the chromatin structure. The chromatin density of cat spermatozoa was significantly larger in case of cells with abnormally changed heads (Hingst et al., 1995). The shape of the spermatozoon head also determines its movement. According to Malo et al. (2006), spermatozoa with elongated heads are faster than spermatozoa with rounded heads.

Compared to silver fox, sperm from Arctic fox had also longer tails (by about 0.04 μm for the blue colour morph and by 1.56 μm for the white colour morph) (Andraszek et al., 2016). It is possible that spermatozoa with longer tails are more likely to reach an egg and are more competitive compared with other spermatozoa (Fitzpatrick and Lüpold, 2014), although long sperm appear to be more motile they deplete their energy faster and have reduced longevity. In contrast to them, shorter sperm live longer (Helfenstein et al., 2010).

There were significant differences between colour morphs of Arctic fox in terms of morphometric characteristics of spermatozoa and also sperm concentration. Sperm concentration in ejaculates from blue foxes was four-times higher compared to white foxes, but sperm dimensions were significantly smaller, both considering the total sperm length and the length of the head and tail. Hirai et al. (2001) suggested that more fertile boars produce ejaculates containing sperm with smaller and shorter heads. On the other hand, Gomendio and Roldan (1991) investigated the dimensions of mammalian sperm and observed that longer sperm cells may be an adaptation that increases sperm competitiveness. Studies revealed that there is some reproductive potential within which sperm cell size can be compensated by their concentration (Gomendio and Roldan, 2008; Tourmente at al., 2011).

A limited number of studies investigated the relationship between sperm concentration and the size and shape of spermatozoa. Rijsselaere investigated dog semen (2004) and reported that in ejaculates with lower sperm concentration (approx. 50x10^6^/cm^3^) sperm heads were shorter and thinner, and the tails were longer compared to ejaculates with sperm concentration of 200x10^6^/cm^3^. Studies on bull semen demonstrated that in less concentrated ejaculates the sperm head perimeter was greater compared to ejaculates with higher sperm concentration (Kondracki et al., 2012). Ejaculates with low sperm cell concentration collected from stallions contained sperm with greater perimeter and sperm head area but also longer tails (Waheed et al., 2015). Less concentrated ejaculates from Arctic foxes contained sperm cells with significantly longer and wider heads, which also corresponded with greater perimeter and sperm head area compared to sperm cells from more concentrated ejaculates. Perhaps the shape and size of male sex cells, depend on the concentration of spermatozoa in the ejaculate. Contrary to the reports of other authors, the apparent relationship was discovered to be statistically insignificant for both blue and white Arctic foxes (Rijsselaere et al., 2004; Kondracki et al., 2006). On the other hand, the statistically significant correlation coefficients between the physical characteristics of Arctic fox ejaculates and the morphometric parameters of spermatozoa were confirmed in previous own research by the author (Stasiak et al., 2019). Our study demonstrated that the most common morphological anomaly of sperm from male Arctic foxes was the strongly coiled sperm tail, called the Dag defect. This defect may be a consequence of abnormal spermatogenesis during sperm maturation.

## Conclusions

In conclusion, the dimensions of spermatozoa from Arctic foxes depend on the male colour morphs. Spermatozoa from blue Arctic foxes were significantly smaller (smaller heads and shorter tails) than those from white Arctic foxes. It is probably related to the concentration of sperm in the ejaculates. Blue Arctic foxes produced ejaculates with significantly higher concentration and total number of spermatozoa compared to white Arctic foxes. The differences shown between particular sperm dimensions indicate the occurrence of gametes differing in shape depending on the sperm concentration. Less concentrated ejaculates contained sperm characterized by larger dimensions compared to sperm from more concentrated ejaculates.
